# Prevalence and Determinants of Viral Suppression in Young People Living with HIV on Antiretroviral Therapy in Southern Africa: A Cross-Sectional Analysis of HIV Survey Data of 2020 and 2021

**DOI:** 10.1007/s10461-025-04662-6

**Published:** 2025-03-03

**Authors:** Takondwa Charles Msosa, Alinune Kabaghe, Hussein Hassan Twabi, Samuel Mpinganjira, Wongani Mzumara, Marion Sumari-De Boer, Rob Aarnoutse, Tobias Rinke De Wit, Geoffrey Chipungu, Kennedy Ngowi, Newton Kalata, Madalo Mukoka, Chisomo Msefula, Iraseni Swai, Boston Zimba, Robina Semphere, Glory Makhumba, Marriott Nliwasa

**Affiliations:** 1 Amsterdam UMC, Department of Global Health, Location University of Amsterdam, Amsterdam, The Netherlands; 2 Helse Nord Tuberculosis Initiative, Department of Pathology, Kamuzu University of Health Sciences, Blantyre, Malawi; 3 Division of Global HIV and TB, US Centers for Disease Control and Prevention, Lilongwe, Malawi; 4 Department of Global Health, University of Washington, Seattle Campus, US; 5 Department of HIV and AIDS, Ministry of Health, Lilongwe, Malawi; 6 Kilimanjaro Clinical Research Institute, Moshi, Tanzania; 7 Department of Pharmacy, Radboud University Medical Center, Research Institute for Medical Innovation, Nijmegen, The Netherlands; 8 Amsterdam Institute for Global Health and Development, Amsterdam, The Netherlands; 9 The World Health Organisation, Malawi Country Office, Lilongwe, Malawi

**Keywords:** HIV, Viral suppression, Young people living with HIV, ART, Southern Africa

## Abstract

Young People Living with HIV (YPLHIV, 15–24 years) are an important demographic of Persons Living with HIV (PLHIV) globally and in Southern Africa. However, YPLHIV experience poor outcomes across the HIV diagnostic and treatment cascade due to multiple factors. We estimated the prevalence and determinants of HIV viral suppression in YPLHIV on antiretroviral therapy (ART) in selected Southern African countries. We used publicly available data from Malawi, Zimbabwe, Mozambique, Lesotho, and Eswatini collected during the Population-based HIV Impact Assessments (PHIAs) of 2020 to 2021. Weighted proportions, and 95% confidence intervals (CI) were computed to estimate the prevalence of viral suppression (< 1000c/ml) and bivariate and multivariate analyses were conducted to identify determinants of viral suppression. A total of 855 records of YPLHIV on ART were included in the analysis. The prevalence of viral suppression in YPLHIV on ART was 82.4% (95% CI: 76.7, 86.9). Residing in Mozambique and duration on ART were inversely associated with viral suppression; adjusted odds ratios (AORs) of 0.37 (95% CI: 0.14, 0.95), and 0.87 (95% CI: 0.80, 0.94), respectively. A negative result in the depression screen, being married/cohabitating, and ever switching an ART regimen were positively associated with viral suppression: AORs of 5.78 (95% CI: 2.21, 15.11), 3.72 (95% CI: 1.44, 9.63), and 3.44 (95% CI: 1.69, 7), respectively. YPLHIV had suboptimal viral suppression lower than the UNAIDS 95% targets and may benefit from further research and tailored interventions addressing modifiable factors associated with viral suppression such as depression.

**Supplementary Information:**

The online version contains supplementary material available at 10.1007/s10461-025-04662-6.

## Introduction

Young people living with HIV (YPLHIV, 15–24 years) constitute a significant and important proportion (8.2%) of Persons Living with HIV (PLHIV). Globally, as of 2022, there were approximately 3,200,000 YPLHIV and the majority, 1,900,000, resided in East and Southern Africa [1]. As countries move towards the UNAIDS’s 95-95-95 targets, which are testing and treatment targets for all subpopulations, age groups and geographic settings [2], it is important to ensure that YPLHIV who are on antiretroviral therapy (ART) achieve viral suppression to improve treatment outcomes and reduce the risk of horizontal and vertical transmission, which is important for epidemic control [3–5]. YPLHIV are a critical demographic because acquiring HIV at a young age increases the potential for HIV transmission over their lifetime. Therefore, achieving viral suppression in YPLHIV on ART is important to reduce HIV transmission [6].

Despite the scale up of ART, many barriers across the HIV diagnosis to treatment cascade have been identified which negatively impact treatment outcomes in YPLHIV. Multiple studies have shown that YPLHIV are the least adherent to ART as compared to older age groups due a variety of factors associated with their formative phase, a period with significant biological, psychological, and social transitions, made more difficult by the addition of an HIV diagnosis [7, 8]. Subsequently, suboptimal treatment adherence puts them at risk of viral non-suppression and HIV associated morbidity and mortality: HIV/AIDS is one of the leading causes of mortality among adolescents in sub-Saharan Africa [9].

Therefore, for countries to achieve epidemic control, eradicate the HIV/AIDS pandemic, and improve treatment outcomes, interventions need to be designed and implemented to ensure that YPLHIV on ART achieve viral suppression [10–12]. Understanding factors associated with viral suppression in YPLHIV on ART is critical to provide evidence for designing effective interventions and policies to improve viral suppression in this demographic [13, 14].

Most studies that describe determinants of viral suppression in YPLHIV use routine clinic or facility level data. However, regional level data could provide useful information and insights that are representative and generalisable to a larger context. Furthermore, since a wide range of important variables are collected in HIV surveys, this was an opportunity to explore potential relationships that are of clinical and public health importance. Therefore, to estimate viral suppression prevalence and determinants in Southern Africa, we conducted a pooled analysis of HIV surveys from Malawi, Mozambique, Zimbabwe, Eswatini, and Lesotho that were conducted between 2020 and 2021. These countries were selected due to their comparable HIV burden, geographical proximity, similarities in their sociocultural and economic characteristics, and that the surveys were conducted in the same years.

## Methods

We analysed data from Population-based HIV Impact Assessments (PHIAs) of Malawi, Mozambique, Zimbabwe, Lesotho, and Eswatini. PHIAs are cross-sectional, two stage, cluster-randomised nationally representative household surveys. The objectives of the surveys were to estimate the subnational prevalence and incidence of HIV, and viral suppression prevalence. The details on the methodology and sampling strategy of the surveys have been described in detail elsewhere [15–19]. For our analyses, we included all YPLHIV that were determined to be on ART either self-reported or through blood tests of ART metabolites.

Since our analysis required the merging of datasets from surveys conducted in different countries, we used the concatenation method to merge the datasets and their respective survey weights [20]. This method involved identifying the country with the largest number of replicate weights, randomly assigning the replicate weights of the other datasets, and filling in the missing weights with the full survey weight for each record [20]. To estimate variance, we used the Jack-knife variance estimation method. All analyses took into consideration the survey design.

Our outcomes were the prevalence and determinants of HIV viral suppression in YPLHIV on ART at a cut-off of less than 1000 copies/ml. This cut-off was selected as there is strong evidence that individuals who have suppressed viral loads defined at this cut-off have an almost zero risk of horizontal transmission which is important for epidemic control [10]. We also estimated the prevalence of viral suppression at a cut-off of < 200 copies/ml as there is strong evidence of zero transmission risk through homosexual or heterosexual sexual intercourse [11, 12]. In addition to estimating viral suppression prevalence in YPLHIV on ART, we compared the prevalence of viral suppression in YPLHIV on ART with older age groups: 25–34, 35–44, 45–54, 55–64, 65 years and older using the same merged dataset.

A priori potential determinants of viral suppression in YPLHIV were based on existing literature, prior knowledge of the subject, and the variables available in the dataset. These were country of residence, sex, age, residence, marital status, education completed, depression screen result (as per the Patient Health Questionnaire-2 tool), anxiety screen result (as per the Generalised Anxiety Disorder 2-item tool), alcoholism screen result (as per the Alcohol Use Disorders Identification Test tool), disclosure to family, disclosure to friends, wealth quintile, ART clinic travel time, ART clinic travel difficulties, years on ART, ever switched ART regimen, and self-reported adherence ( > = 95%). More information on the variables and categorisations described in detail in Supplemental Material 1.

Weighted proportions, and 95% confidence intervals were computed to estimate the prevalence of viral suppression in YPLHIV on ART. Furthermore, to identify predictors of viral suppression, we conducted bivariate analyses of each predictor and the primary outcome (Table 1). For categorical predictors, data were summarised using weighted frequencies and proportions, we then used Chi-squared tests with Rao & Scott’s second-order correction for hypotheses testing. For continuous variables, medians and interquartile ranges (IQRs) were used to summarise the data, and we used Wilcoxon rank-sum tests for complex survey samples for hypothesis testing. Variables that had a p-value of less than 0.2 in the bivariate analyses were then specified in the multivariable logistic regression model referred to as the “full model”. We used a higher p-value threshold to identify determinants to be specified in the full model because some variables could have been classified as non-significant in the bivariate analysis due to confounding [21]. Furthermore, we used this approach to maintain a reasonable sample size due to missingness in some variables (Table 1).

The full model was adjusted for sex, age, and country of residence regardless of the hypothesis test results of these variables in the bivariate analyses. We also conducted backwards variable selection to specify a parsimonious model, referred to as the “reduced model” in this paper. The Wald test was used for hypothesis testing in the models with the null hypothesis that all coefficients associated with a particular regression term are zero. This was used to describe the overall importance of a variable and for backwards variable selection to specify the reduced model. However, all interpretations and conclusions were based on the full model, and selection and interpretations of significant predictors of viral suppression were based on post adjustment confidence intervals in the full model. Statistical significance was defined as a p-value < 0.05. A complete case analysis was conducted.

### Ethical Considerations

The PHIA surveys were reviewed by CDC, deemed not research, and were conducted consistent with applicable federal law and CDC policy^1^. In addition, the institutional review boards for each country and the implementing partners reviewed and approved the protocols.

## Results

### Inclusion

**Fig. 1:**
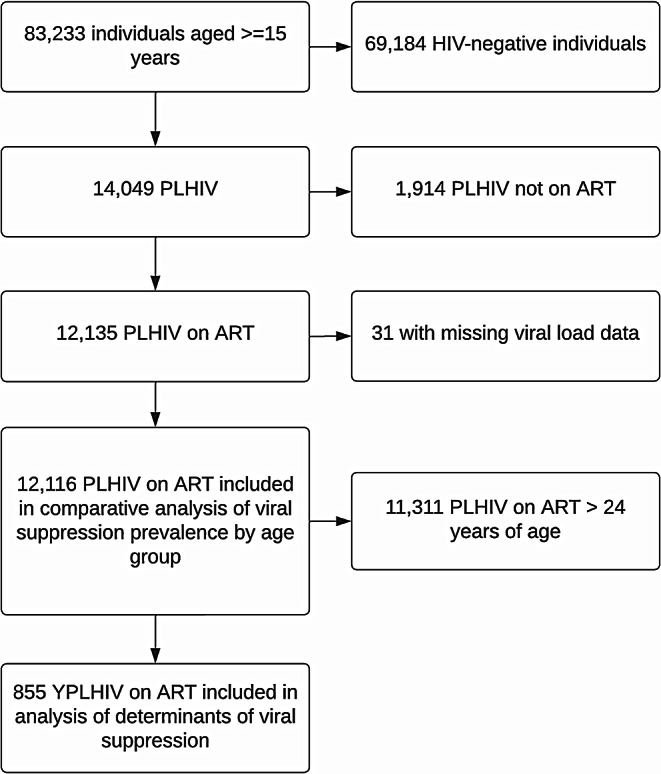
Flowchart of participants included in the analyses of the nationally representative HIV surveys of Malawi, Mozambique, Lesotho, Eswatini, and Zimbabwe of 2021–2021 (*PLHIV = Persons Living with HIV, ART = Antiretroviral Therapy)

A total of 83,233 individuals, 15 year and older, participated in the surveys that occurred between 2020 and 2021. For our primary analysis, we had a total of 855 YPLHIV on ART weighted to a population of 349,309 YPLHIV on ART (Fig. 1).

### Demographic and Clinical Characteristics

The distribution of participants by country of residence was as follows: Eswatini (*n* = 206, 4.0%), Lesotho (*n* = 194, 4.8%), Malawi (*n* = 147, 17.0%), Mozambique (*n* = 128, 49.5%), and Zimbabwe (*n* = 180, 24.6%). In terms of sex, there were 187 males (25.1%) and 668 females (74.9%). The median age of participants was 21 years with an interquartile range (IQR) of 18 to 22 years. More information on participant and clinical characteristics including the results of bivariable analyses can be seen in Table 1.

**Table 1:** Sociodemographic, clinical characteristics, and bivariate analyses

Variable	Overall (*N* = 855)^1 n (%)^	Suppressed, *N* = 720^2, 3 n (%)^	Not suppressed, *N* = 135^2, 3 n (%)^	*P*-value^4^
**Country**				
Eswatini	206 (4.0)	185 (89.4)	21 (10.6)	F = 2.71, *p* = 0.06
Lesotho	194 (4.8)	158 (81.3)	36 (18.8)	
Malawi	147 (17.0)	132 (90.7)	15 (9.3)	
Mozambique	128 (49.5)	100 (79.1)	28 (20.9)	
Zimbabwe	180 (24.6)	145 (81.0)	35 (19)	
**Sex**				
Male	187 (25.1)	149 (80.0)	38 (20)	F = 0.34, *p* = 0.56
Female	668 (74.9)	571 (82.7)	97 (17.3)	
**Age (median [IQR])**	21.00 [18.0, 22.0]	21.0 [19.0, 23.0]	20.00 [18.0, 22.0]	t = 2.05, *p* = 0.04
**Residence**				
Rural	530 (60.8)	454 (82.3)	76 (17.7)	F = 0.03, *p* = 0.86
Urban	325 (39.3)	266 (81.6)	59 (18.4)	
**Marital status**				
Divorced/separated/widowed	108 (17.1)	88 (75.7)	20 (24.3)	F = 2.13, *p* = 0.12
Married/living together	260 (35.3)	231 (87.8)	29 (12.2)	
Never married	483 (47.6)	397 (80)	86 (20)	
Missing	4	4	0	
**Highest education completed**				
No education/Primary	356 (51.2)	293 (78.8)	63 (21.2)	F = 3.25, *p* = 0.07
Secondary/tertiary	496 (48.9)	425 (85.5)	71 (14.5)	
Missing	3	2	1	
**Depression screen**				
Positive	53 (17.6)	38 (62.6)	15 (37.4)	F = 6.76, *p* < 0.05
Negative	794 (92.4)	677 (83.7)	117 (16.4)	
Missing	8	5	3	
**Anxiety screen**				
Positive	46 (5.5)	36 (79.3)	10 (20.7)	F = 0.17, *p* = 0.68
Negative	800 (94.5)	679 (82.2)	121 (17.8)	
Missing	9	5	4	
**Alcohol use screen**				
Hazardous drinking	53 (6.3)	44 (84.6)	9 (15.4)	F = 0.09, *p* = 0.77
no drinking/not hazardous	800 (93.7)	674 (81.9)	126 (18.1)	
Missing	2	2	0	
**Disclosure to family**				
Disclosed	632 (75.4)	532 (81.3)	100 (18.7)	F = 0.32, *p* = 0.57
Not disclosed	140 (24.6)	122 (84.4)	18 (15.6)	
Missing	83	66	17	
**Disclosure to friend**				
Disclosed	105 (9.4)	84 (77.5)	21 (22.5)	F = 0.50, *p* = 0.48
Not disclosed	667 (90.6)	570 (82.5)	97 (17.5)	
Missing	83	66	17	
**Wealth quintile**				
Lowest	170 (17.7)	143 (86.1)	27 (13.9)	F = 0.77, *p* = 0.54
Second	169 (24.2)	140 (83.6)	29 (16.4)	
Middle	171 (16.8)	143 (76)	28 (24)	
Fourth	170 (18.1)	147 (79)	23 (21)	
Highest	171 (23.1)	143 (84.1)	28 (16)	
Missing	4	4	0	
**ART clinic travel time**				
1 h to 2 h	185 (21.9)	163 (88.0)	22 (12.0)	F = 0.79, *p* = 0.50
30 min to 1 h	222 (33.7)	187 (82.2)	35 (17.8)	
Less than 30 min	262 (32.9)	215 (78.9)	47 (21.1)	
More than 2 h	94 (11.5)	81 (79.2)	13 (20.8)	
Missing	92	74	18	
**ART clinic travel difficulties**				
Difficulties	168 (25.1)	146 (83.9)	22 (16.1)	F = 0.21, *p* = 0.65
No difficulties	605 (74.9)	508 (81.4)	97 (18.6)	
Missing	82	66	16	
**Ever switched ART regimen**				
Not switched	426 (54.4)	351 (75.9)	75 (24.1)	F = 10.19, *p* < 0.01
Switched	331 (45.6)	292 (89.5)	39 (10.5)	
Missing	98	77	21	
Years on ART (median [IQR])	2.0 [1.0, 6.0]	2.0 [1.0, 5.0]	4.0 [1.0, 9.0]	t = -2.11, *p* = 0.04
Missing	112	91	21	
**Self-reported adherence to ART ( > = 95%)**				
Adherent	613 (81.0)	524 (82.6)	89 (17.4)	F = 0.24, *p* = 0.62
Not adherent	151 (19.0)	121 (79.6)	30 (20.4)	
Missing	91	75	16	

^1^ unweighted n (weighted column %); Median (IQR)

^2^ unweighted n (weighted row %); Median (IQR)

^3^viral suppression was defined < 1000 copies/ml

^4^ chi-squared test with Rao & Scott’s second-order correction; Wilcoxon rank-sum test for complex survey samples; variables with a p-value < 0.2 were specified in the multivariable logistic regression model

### Prevalence of Viral Suppression in YPLHIV on ART

Using a cut-off of < 1000 copies/ml, we estimated that the prevalence of viral suppression in YPLHIV on ART was 82.4% (95% CI: 76.7–86.9). The prevalence was much lower than older PLHIV (F = 5.82, *p* < 0.001) who had a viral suppression point prevalence of 90% or higher as can be seen in Table 2.

**Table 2:** Prevalence of viral suppression (< 1000c/ml) by age group

Age group	Prevalence of viral suppression % (95% CI)	*P* value^1^
15–24 years	82.4 (76.7–86.9)	F = 5.82, *p* < 0.001
25–34 years	90.3 (87.2–92.6)	
35–44 years	92.8 (90.9–94.4)	
45–54 years	96.1 (94.5–97.3)	
55–64 years	96.6 (94.4–98)	
65 + years	94.8 (90.3–97.3)	

^1^chi-squared test with Rao & Scott’s second-order correction

**Fig. 2:**
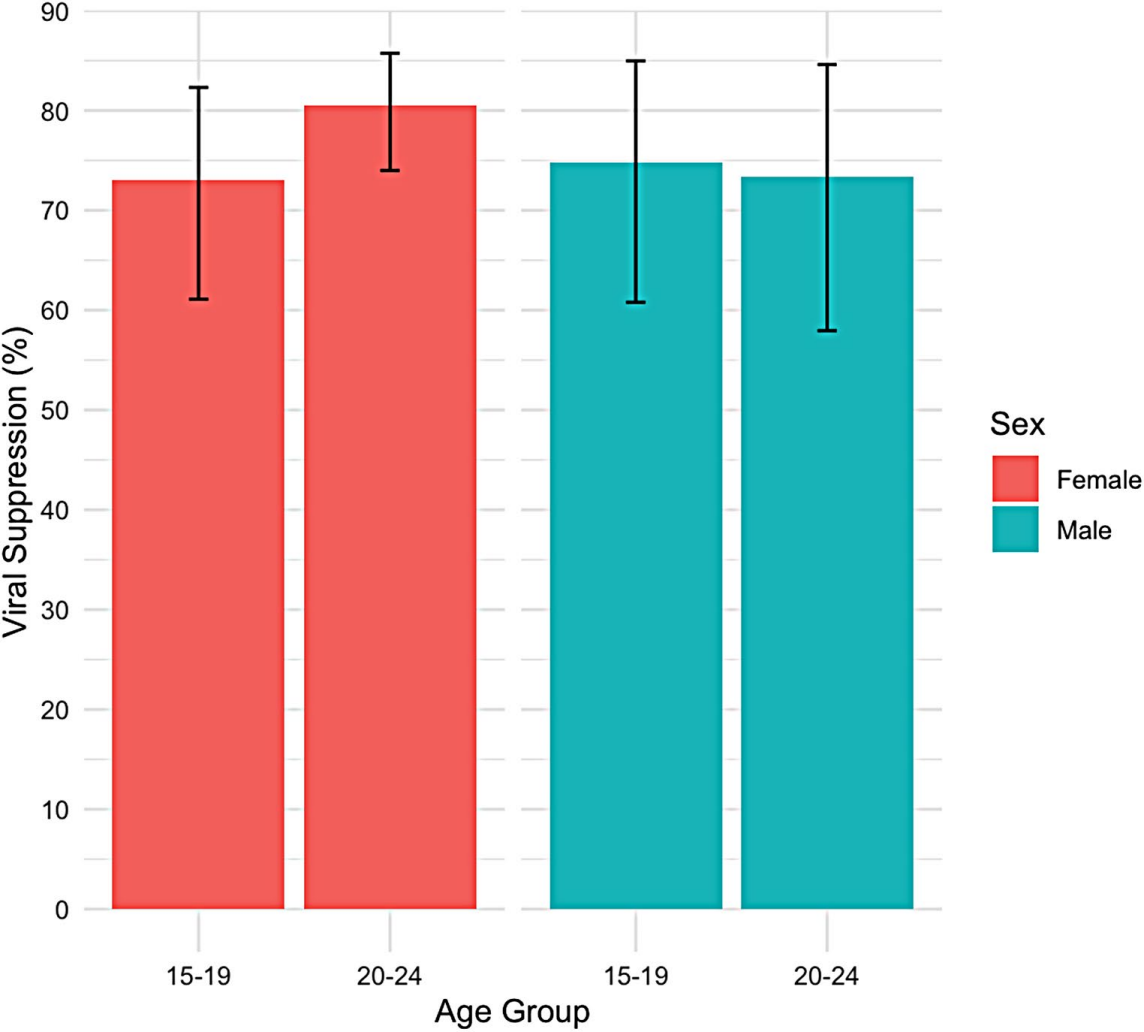
Viral Suppression (< 1000c/ml) in YPLHIV disaggregated by age and sex

There was no significant difference in viral suppression among YPLHIV by age and sex. Among females, viral suppression prevalence was 78.8% (95% CI: 66–82.3) for ages 15–19 and 84.5% (95% CI: 78.6–88.9) for ages 20–24 (F = 1.62, *p* = 0.20). Similarly, among males, prevalence was 77.3% (95% CI: 63.3–87.1) for ages 15–19 and 83% (95% CI: 70.6–90.8) for ages 20–24 (F = 0.03, *p* = 0.87). Further details can be seen in Fig. 2.

### Determinants of Viral Suppression in YPLHIV

In addition to the predetermined variables: age, and sex, six variables were specified in the multivariable logistic regression model based on the results of the bivariate analyses in Table 1. These variables were country of residence, marital status, highest education completed, depression screen, years on ART, and ever switching an ART regimen. Table 3 reports the crude and adjusted odds ratios for the variables included in the full model.

In terms of country of residence, YPLHIV showed varying odds of viral suppression with Eswatini as the reference. For those from Lesotho, Malawi, and Zimbabwe, there was no statistically significant difference in viral suppression when compared to Eswatini. However, participants from Mozambique had significantly lower odds of viral suppression in relation to Eswatini, with an AOR of 0.37 (95% CI: 0.14, 0.95).

YPLHIV on ART who were married or cohabiting had significantly higher odds of viral suppression compared to those who were divorced, separated, or widowed, with an AOR of 3.72 (95% CI: 1.44, 9.63). In contrast, those who had never married did not show a significant difference in odds of viral suppression compared to the reference group with an AOR of 1.19 (95% CI: 0.45, 3.17).

The highest education completed showed a non-significant relationship with viral suppression when comparing YPLHIV with secondary or tertiary education with those with no education or primary education; AOR of 1.65 (95% CI: 0.80, 3,40).

Depression status was significantly associated with viral suppression. Participants who did not screen positive for depression had significantly higher odds of viral suppression compared to those who had a positive depression screen with an AOR of 5.78 (95% CI: 2.21, 15.11).

The duration of time participants had been on antiretroviral therapy (ART) was inversely associated with viral suppression. Longer duration on ART was associated with lower odds of viral suppression, with an AOR of 0.87 (95% CI: 0.80, 0.94) per additional year on ART. Participants who had switched their ART regimen had significantly higher odds of viral suppression compared to those who had not switched, with an AOR of 3.44 (95% CI: 1.69, 7).

Lastly, age and sex did not significantly affect the odds of viral suppression after adjustment. There was no statistically significant difference in the odds of viral suppression when comparing females to males with AOR of 1.29 (95% CI: 0.61, 2.74). A unit increase in age was not significantly associated with viral suppression with an AOR of 0.86 (95% CI: 0.84, 1.10).

**Table 3:** Full multivariable logistic regression model of odds of viral suppression in relation to non-suppression (< 1000 copies/ml

Characteristic	Crude Odds Ratio (95% Confidence Interval)	Adjusted Odds Ratio (95% Confidence Interval)	*P*-value*
**Country**			
Eswatini	Ref	Ref	F = 2.33, *p* = 0.06
Lesotho	0.51 (0.28, 0.93)	0.49 (0.23, 1.04)	
Malawi	1.16 (0.50, 2.68)	1.34 (0.46, 3.93)	
Mozambique	0.45 (0.23, 0.88)	0.37 (0.14, 0.95)	
Zimbabwe	0.51 (0.26, 0.97)	0.70 (0.31, 1.58)	
**Age**	1.10 (1.0, 1.21)	0.86 (0.84, 1.10)	F = 0.23, *p* = 0.63
**Sex**			
Male	Ref	Ref	F = 0.46, *p* = 0.50
Female	1.19 (0.65, 2.17)	1.29 (0.61, 2.74)	
**Marital Status**			
Divorced/separated/widowed	Ref	Ref	F = 4.35, *p* < 0.05
Married/cohabiting	2.32 (0.95, 5.63)	3.72 (1.44, 9.63)	
Never married	1.28 (0.61, 2.71)	1.19 (0.45, 3.17)	
**Education**			
No education/Primary	Ref	Ref	F = 1.88, *p* = 0.17
Secondary/tertiary	1.58 (0.96, 2.61)	1.65 (0.80, 3.40)	
**Depression screen**			
Positive	Ref	Ref	F = 12.98, *p* < 0.001
Negative	3.06 (1.27, 7.39)	5.78 (2.21, 15.11)	
**Years on ART**	0.92 (0.86, 0.98)	0.87 (0.80, 0.94)	F = 12.81, *p* < 0.001
**Ever switched ART**			
Not switched	Ref	Ref	F = 11.72, *p* < 0.001
Switched	2.7 (1.43, 5.12)	3.44 (1.69, 7)	

*Wald’s Test of adjusted odds ratio; p-value refers to the AOR

In the reduced multivariable logistic regression model, depression screen result, years on ART, and ever switched ART regimen were the most important determinants of viral suppression, as can be seen in Table 4.

**Table 4:** Reduced multivariable logistic regression model of odds of viral suppression in relation to non-suppression (< 1000 copies/ml)

Characteristic	Crude Odds Ratio (95% Confidence Interval)	Adjusted Odds Ratio (95% Confidence Interval)	*P*-value*
**Depression screen**			
Positive	Ref	Ref	F = 10.98, *p* < 0.01
Negative	3.06 (1.27, 7.39)	5.51 (2, 5.23)	
**Years on ART**	0.92 (0.86, 0.98)	0.88 (0.82, 0.94)	F = 12.53, *p* < 0.001
**Ever switched ART**			
Not switched	Ref	Ref	F = 16.14, *p* < 0.001
Switched	2.7 (1.43, 5.12)	4.02 (2.03, 7.95)	

*Wald’s Test of adjusted odds ratio

### Additional Analysis of Viral Suppression (< 200 copies/ml)

Using a cut-off of < 200 copies/ml, we estimated that the prevalence of viral suppression in YPLHIV on ART was 77.16% (95% CI: 72.13 − 81.52%) not significantly different from the higher threshold of < 1000 copies/ml which estimated the prevalence of viral suppression to be 82.4% (95% CI: 76.7–86.9).

Using the same model building procedure, significant determinants of viral suppression were mostly consistent at this threshold when compared to the < 1000 copies/ml threshold as can be seen in Table 5. These determinants were country of residence, marital status, depression screen result, years on ART, and ever switching an ART regimen. Age, sex, and self-reported adherence had no significant relationship on viral suppression at this threshold as well. More information on bivariate analyses and the reduced model at this cut-off can be seen in Supplemental Material 2.

**Table 5:** Full multivariable logistic regression model of odds of viral suppression in relation to non-suppression (< 200 copies/ml)

Characteristic	Crude Odds Ratio (95% Confidence Interval)	Adjusted Odds Ratio (95% Confidence Interval)	*P*-value*
**Country**			
Eswatini	Ref	Ref	F = 3.74, *p* < 0.05
Lesotho	0.48 (0.29, 0.80)	0.40 (0.21, 0.77)	
Malawi	0.94 (0.47, 1.88)	0.91 (0.37, 2.21)	
Mozambique	0.45 (0.25, 0.83)	0.31 (0.14, 0.69)	
Zimbabwe	0.61 (0.34, 1.07)	0.70 (0.34, 1.44)	
**Age**	1.09 (1.0, 1.19)	0.98 (0.87, 1.11)	F = 0.08, *p* = 0.78
**Sex**			
Male	Ref	Ref	F = 0.03, *p* = 0.87
Female	1.26 (0.71, 2.22)	1.07 (0.48, 2.39)	
**Marital status**			
Divorced/separated/widowed	Ref	Ref	F = 5.29, *p* < 0.05
Married/living together	2.60 (1.15, 5.89)	3.41 (1.34, 8.66)	
Never married	1.31 (0.66, 2.59)	0.77 (0.30, 1.96)	
**Completed education**			
No education/Primary	Ref	Ref	F = 3.66, *p* = 0.06
Secondary/Tertiary	1.72 (1.05, 2.84)	1.95 (0.98, 3.86)	
**Depression screen**			
Depression	Ref	Ref	F = 12.91, *p* < 0.001
No depression	2.84 (1.19, 6.75)	5.18 (2.10, 12.78)	
**Years on ART**	0.94 (0.88, 1.00)	0.90 (0.84, 0.97)	F = 7.60, *p* < 0.05
**Ever switched ART regimen**			
Never switched	Ref	Ref	F = 7.15, *p* < 0.05
Ever switched	1.96 (1.14, 3.37)	2.33 (1.25, 4.35)	
**Self-reported Adherence ( > = 95%)**			
Adherent	Ref	Ref	F = 0.91, *p* < 0.34
Not adherent	0.59 (0.31, 1.13)	0.71 (0.35, 1.44)	

*Wald’s Test of adjusted odds ratio

## Discussion

Our analysis showed that YPLHIV on ART had a viral suppression prevalence of 82.4% (95% CI: 76.7, 86.9). Additionally, using a more ambitious viral suppression cut-off of < 200 copies/ml, 77.16% (95% CI: 72.13 − 81.52%) of YPLHIV on ART were suppressed. Furthermore, significant positive determinants of viral suppression were being married or cohabiting, not having depression, and having switched ART regimens, when compared to those that were divorced, separated, widowed; depressed; and those that never switched regimens respectively. Conversely, residing in Mozambique and having a longer duration on ART were associated with lower odds of viral suppression.

Our analysis revealed that the prevalence of viral suppression in YPLHIV on ART was sub-optimal and below the current UNAIDS 95-95-95 targets and the UNAIDS 90-90-90 targets at the time. Our findings are consistent with other studies in the region on viral suppression in YPLHIV on ART which showed a similar trend [7, 22, 23]. YPLHIV on ART may benefit from priority care including screening and detection of viral non-suppression due to either ART failure or HIV drug resistance. Furthermore, differentiated service delivery models may address psychological and social barriers of YPLHIV to access HIV care and treatment [24, 25].

Our findings on determinants of viral suppression in YPLHIV on ART are informative and consistent with other literature. First, screening of mental health conditions like depression in HIV Care and Treatment (HCT) clinics may be employed to identify YPLHIV who are more likely to experience treatment failure. Evidence from similar contexts has shown that a diagnosis of a mental health disorder is strongly associated with treatment failure or viral non suppression [26]. Therefore, the World Health Organisation (WHO) has prioritised the integration of mental health and HIV interventions to improve the quality of life of PLHIV and optimise treatment outcomes [27]. PLHIV have higher prevalence of mental health disorders when compared to the general population which adversely affects treatment compliance and outcomes [27–29]. Therefore, screening for mental health disorders like depression in ART clinics could be effective in identifying YPLHIV that are not suppressed or are at risk of developing treatment failure. However, more evidence is needed to understand the feasibility, acceptability, and effectiveness of such screening programs in high burden low resource settings. Multifaceted approaches that combine direct mental health interventions, family involvement, peer support, and attention to social determinants are essential for improving the mental health of YPLHIV [30, 31]. However, these interventions should be feasible, and acceptable in high burden low-income settings taking advantage of available lay cadres due to the limited availability of mental health practitioners [31]. For example, innovative approaches such as group support psychotherapy delivered by lay cadres have been explored to enhance mental wellbeing and adherence to ART among YPLHIV [32, 33]. Telehealth and mobile health (mHealth) technologies also present new avenues for delivering scalable mental health interventions for YPLHIV, particularly in low-resource settings where access to healthcare and human resources may be limited [34, 35]. These technologies can facilitate ongoing engagement and provide timely mental health resources tailored to the unique and complex needs of YPLHIV.

Second, our results have shown that the increase in the duration on ART in YPLHIV was negatively associated with viral suppression which is consistent with other studies in the region [36]. Therefore, even though evidence shows that the majority of PLHIV achieve viral suppression within one year of initiating ART, our findings including other evidence show that the relationship is more complex and might evolve with increasing duration on ART and as individuals stay longer in care [37]. Additional studies may help to understand whether this phenomenon is due to the development of drug resistance, treatment fatigue, mental health disorders or other factors that might negatively affect adherence and viral suppression as individuals are progressing through care [38–40]. Moreover, as YPLHIV remain in care for extended periods, ART clinics may help ensure medication adherence by adapting treatment and psychological support at every stage of their treatment journey. Additionally, optimising and expanding routine and targeted viral load monitoring among YPLHIV on ART may contribute to assessing treatment response regularly.

Third, our results have shown that ever switching an ART regimen was positively associated with the odds of viral suppression. Therefore, even though data was not available on the reasons for switching and the regimens involved; our findings could be a signal that expanding regimen switching to more potent and adherence forgiving regimens like dolutegravir based regimens, which were being rolled out at the time [41, 42], complemented by increasing the coverage of HIV drug resistance testing, adherence monitoring, and support could improve viral suppression in this demographic [12–14].

Fourth, our results showed that there is heterogeneity of viral suppression in YPLHIV on ART among the countries that were included in the analysis. This could be due to country specific factors that could not be explored in this analysis. However, developing country specific evidence informed interventions may improve treatment outcomes in YPLHIV in each country.

Lastly, being married or cohabitating with a partner was positively associated with viral suppression compared to those who were divorced, separated, or widowed. While limited data are available on the relationship between marital status and viral suppression in YPLHIV on ART, a similar study involving adults living with HIV in the same countries did not find an association with viral suppression [26]. Additional research could help clarify this relationship in YPLHIV on treatment and how interventions may be optimised to tailor to the relationship status of YPLHIV.

Our study had two main limitations. First, the main objective of the PHIA surveys was to estimate subnational prevalence of HIV in participating countries and not the prevalence of viral suppression in YPLHIV on ART. Therefore, our effective sample size was significantly reduced. This negatively impacted the precision of our estimates and only allowed us to detect large effect sizes as statistically significant. Second, as we were only limited to variables that were collected during the surveys, we could not explore relationships between viral suppression and other important predictors or adjust for confounders that were not collected such as whether HIV transmission was either vertical or horizontal, or presence of HIV drug resistance, to mention a few.

However, our analysis also had several strengths. First, by pooling survey data from similar contexts, we were able to explore predictors of viral suppression in this important demographic which could have been challenging if we only had data of one country, as frequentists methods usually require large sample sizes to adequately explore relationships. Second, our sensitivity analysis showed that predictors of suppression were consistent even at both viral suppression thresholds of < 1000 & < 200 copies/ml, strengthening the validity of our findings.

## Conclusion

In conclusion, our analysis has shown that YPLHIV had sub-optimal viral suppression prevalence. Therefore, evidence informed interventions such as expanding mental health care access and screening programs in low-resource settings for YLPHIV on ART could be implemented to potentially improve viral suppression in this demographic. Furthermore, more studies that specifically explore individual determinants of viral suppression in YPLHIV on ART could be conducted to help understand how they affect viral suppression in this demographic.

## Electronic Supplementary Material

Below is the link to the electronic supplementary material.

Supplementary Material 1

Supplementary Material 2

## Data Availability

The data that support the findings of this study are available from the PHIA website upon reasonable request.

## Footnotes

1 § See e.g., 45 C.F.R. part 46.102(l) [2], 21 C.F.R. part 56; 42 U.S.C. § 241(d); 5 U.S.C. § 552a; 44 U.S.C. § 3501 et seq.
