# Supplementary material for: Prevalence and Determinants of Viral Suppression in Young People Living with HIV on Antiretroviral Therapy in Southern Africa: A Cross-Sectional Analysis of HIV Survey Data of 2020 and 2021
Source: AIDS Behav. 2025 Mar 3;29(6):1962–72. doi: 10.1007/s10461-025-04662-6 (PMC12075269; doi:10.1007/s10461-025-04662-6)
Supplement: Supplementary file 1 — Supplementary Material 1 [file 10461_2025_4662_MOESM1_ESM.docx]

**SUPPLEMENTAL MATERIAL 1: VARIABLE DEFINITIONS**

| **Variable** | **Definition** | **type** | **Levels** |
| --- | --- | --- | --- |
| Country | Country of residence | Categorical | Eswatini, Lesotho, Malawi, Mozambique, Zimbabwe |
| Sex | Self-reported Sex | Categorical | Male; Female |
| Age | Self-reported age in years | Numeric | Not applicable |
| Marital Status | Self-reported marital status | Categorical | Divorced/separated/widowed; Married/living together; Never married |
| Highest education completed | Self-reported highest level of education completed | Categorical | No education/primary; secondary/tertiary |
| Depression screen | Defined by the Patient Health Questionnaire-2 (PHQ-2) tool. A score of 3 or greater was the cut-off for screening positive for depression | Categorical | Depression; no depression |
| Anxiety screen | Defined by the Generalised Anxiety Disorder 2-item (GAD-2) tool. A score of 3 or greater was the cut-off for screening positive for anxiety | Categorical | Anxiety; no anxiety |
| Alcohol use | Defined by Alcohol Use Disorders Identification Test (AUDIT-C) tool. For men, a score of 4 or more is considered positive. For women, a score of 3 or more was considered positive | Categorical | Hazardous drinking; none or not hazardous drinking |
| Region | As defined by household location | Categorical | Rural; Urban |
| Wealth quintile | As defined by the wealth index score | Categorical | Lowest; second; middle; fourth; highest |
| Disclosure to family | Self-reported HIV positive status disclosure to family | Categorical | Disclosed; not disclosed |
| Disclosure to friend | Self-reported HIV positive status disclosure to family | Categorical | Disclosed; not disclosed |
| ART clinic travel time | Self-reported ART clinic travel time | Categorical | Less than 30 minutes; 30 minutes to 1 hour; 1 hour to 2 hours; More than 2 hours |
| Ever switched ART | Self-reported on whether a participant has ever changed an ART regimen | Categorical | Not switched; switched |
| Years on ART | Self-reported years participants have been on ART | Numerical | Not applicable |
| Self-reported adherence (>=95%) | Self-reported whether participants intake 95% or more of prescribed ART in a month | Categorical | Adherent; not adherent |
| HIV viral suppression | Plasma HIV viral load with a cut-off of <1000copies/ml and <200 copies/ml for viral suppression | Categorical | Suppressed; not suppressed |
