# Supplementary material for: Prevalence and Determinants of Viral Suppression in Young People Living with HIV on Antiretroviral Therapy in Southern Africa: A Cross-Sectional Analysis of HIV Survey Data of 2020 and 2021
Source: AIDS Behav. 2025 Mar 3;29(6):1962–72. doi: 10.1007/s10461-025-04662-6 (PMC12075269; doi:10.1007/s10461-025-04662-6)
Supplement: Supplementary file 2 — Supplementary Material 2 [file 10461_2025_4662_MOESM2_ESM.docx]

# Bivariable analysis of viral suppression at <200 copies threshold

| **Variable** | **Overall (N = 854)^1^** | **Suppressed, n = 679^2^** | **Not suppressed, n = 175^2^** | **P-value^3^** |
| --- | --- | --- | --- | --- |
| **Country** |  |  |  |  |
| Eswatini | 206 (4%) | 178 (85.8%) | 28 (14.2%) | F = 2.62, p = 0.06 |
| Lesotho | 194 (4.8%) | 146 (74.4%) | 48 (25.6%) |  |
| Malawi | 146 (17%) | 123 (85%) | 23 (15%) |  |
| Mozambique | 128 (49.5%) | 92 (73.3%) | 36 (26.7%) |  |
| Zimbabwe | 180 (24.6%) | 140 (78.6%) | 40 (21.4%) |  |
| **Sex** |  |  |  |  |
| Male | 187 (25.1%) | 143 (74.1%) | 44 (25.9%) | F = 0.63, p = 0.43 |
| Female | 667 (74.9%) | 536 (78.2%) | 131 (21.8%) |  |
| **Age** | 21 (18, 22) | 21 (19, 23) | 20 (18, 22) | t = 2.34, p = 0.02 |
| **Region** |  |  |  |  |
| Urban | 325 (39.3%) | 247 (76.5%) | 78 (23.6%) | F = 0.06, p = 0.81 |
| Rural | 529 (60.8%) | 432 (77.6%) | 97 (22.4%) |  |
| **Marital status** |  |  |  |  |
| Divorced/separated/widowed | 108 (17.1%) | 80 (68.8%) | 28 (31.2%) | F = 3.53, p = 0.03 |
| Married/cohabiting | 259 (35.3%) | 223 (85.1%) | 36 (14.9%) |  |
| Never married | 483 (47.6%) | 372 (74.2%) | 111 (25.8%) |  |
| Missing | 4 | 4 | 0 |  |
| **Wealth quintile** |  |  |  |  |
| Lowest | 170 (17.7%) | 137 (81.1%) | 33 (18.9%) | F = 0.50, p = 0.73 |
| Second | 169 (24.2%) | 130 (77%) | 39 (23%) |  |
| Middle | 170 (16.8%) | 135 (72.3%) | 35 (27.7%) |  |
| Fourth | 170 (18.1%) | 142 (74.1%) | 28 (25.9%) |  |
| Highest | 171 (23.1%) | 131 (80%) | 40 (20.1%) |  |
| Missing | 4 | 4 | 0 |  |
| **Completed education** |  |  |  |  |
| No education/Primary | 356 (51.2%) | 274 (73.1%) | 82 (27%) | F = 4.67, p = 0.03 |
| Secondary/Tertiary | 495 (48.9%) | 403 (82.4%) | 92 (17.6%) |  |
| Missing | 3 | 2 | 1 |  |
| **Depression screen** |  |  |  |  |
| Depression | 53 (7.6%) | 36 (56.8%) | 17 (43.3%) | F = 6.07, p = 0.01 |
| No depression | 793 (92.4%) | 638 (78.8%) | 155 (21.2%) |  |
| Missing | 8 | 5 | 3 |  |
| **Anxiety screen** |  |  |  |  |
| Anxiety | 46 (5.5%) | 33 (67.4%) | 13 (32.6%) | F = 1.35, p = 0.25 |
| No anxiety | 799 (94.5%) | 641 (78%) | 158 (22%) |  |
| Missing | 9 | 5 | 4 |  |
| **Alcohol use screen** |  |  |  |  |
| Harmful use | 53 (6.3%) | 40 (80.3%) | 13 (19.8%) | F = 0.12, p = 0.73 |
| No harmful use | 799 (93.7%) | 637 (77%) | 162 (23.1%) |  |
| Missing | 2 | 2 | 0 |  |
| **Disclosure to family** |  |  |  |  |
| Disclosed | 631 (75.4%) | 500 (76.77%) | 131 (23.2%) | F = 0.32, p = 0.57 |
| Not disclosed | 140 (24.6%) | 115 (80.1%) | 25 (20%) |  |
| Missing | 83 | 64 | 19 |  |
| **Disclosure to friend** |  |  |  |  |
| Disclosed | 105 (9.4%) | 78 (68.3%) | 27 (31.7%) | F = 1.67, p = 0.20 |
| Not disclosed | 666 (90.6%) | 537 (78.6%) | 129 (21.5%) |  |
| Missing | 83 | 64 | 19 |  |
| **ART clinic travel time** |  |  |  |  |
| Less than 30 minutes | 262 (32.9%) | 203 (75.5%) | 59 (24.5%) | F = 0.41, p = 0.74 |
| 30 minutes to 1 hour | 222 (33.7%) | 182 (81.00%) | 40 (19%) |  |
| 1 hour to 2 hours | 184 (21.9%) | 147 (76.9%) | 37 (23.1%) |  |
| More than 2 hours | 94 (11.5%) | 75 (73.5%) | 19 (26.5%) |  |
| Missing | 92 | 72 | 20 |  |
| **ART clinic travel difficulties** |  |  |  |  |
| Difficulties | 167 (25.1%) | 134 (75%) | 33 (25%) | F = 0.30, p = 0.59 |
| No difficulties | 605 (74.9%) | 481 (78.3%) | 124 (21.8%) |  |
| Missing | 82 | 64 | 18 |  |
| **Years on ART** | 2 (1, 6) | 2 (1, 5) | 4 (1, 8) | t = -2.25, p = 0.03 |
| Missing | 112 | 87 | 25 |  |
| **Ever switched ART regimen** |  |  |  |  |
| Never switched | 425 (54.4%) | 330 (72.6%) | 95 (27.3%) | F = 6.33, p = 0.01 |
| Ever switched | 331 (45.6%) | 274 (84%) | 57 (16 %) |  |
| Missing | 98 | 75 | 23 |  |
| **Self-reported Adherence (>=95%)** |  |  |  |  |
| Not adherent | 151 (19%) | 109 (69.2%) | 42 (30.8%) | F = 2.64, p = 0.11 |
| Adherent | 612 (81%) | 498 (79.2%) | 114 (20.7%) |  |
| Missing | 91 | 73 | 19 |  |
| *^1^* n (weighted column %); Median (IQR)  ^2^n (weighted row %); Median (IQR)  ^3^ chi-squared test with Rao & Scott’s second-order correction; Wilcoxon rank-sum test for complex survey samples | | | | |

# Reduced multivariable logistic model at <200c/ml threshold

| **Characteristic** | **Crude Odds Ratio (95% Confidence Interval)** | **Adjusted Odds Ratio (95% Confidence Interval)** | **P-value*** |
| --- | --- | --- | --- |
| **Country** |  |  |  |
| Eswatini | Ref | Ref | F = 3.66, p = 0.01 |
| Lesotho | 0.5 (0.3, 0.8) | 0.5 (0.3, 0.9) |  |
| Malawi | 0.9 (0.5, 1.9) | 0.7 (0.3, 1.7) |  |
| Mozambique | 0.5 (0.3, 0.8) | 0.27 (0.12, 0.60) |  |
| Zimbabwe | 0.61 (0.34, 1.07) | 0.77 (0.39, 1.54) |  |
| **Marital status** |  |  |  |
| Divorced/separated/widowed | Ref | Ref | F = 5.32, p = 0.01 |
| Married/living together | 2.60 (1.15, 5.89) | 3.05 (1.27, 7.30) |  |
| Never married | 1.31 (0.66, 2.59) | 0.94 (0.42, 2.09) |  |
| **Depression screen** |  |  |  |
| Depression | Ref | Ref | F = 15.71, p <0.001 |
| No depression | 2.84 (1.19, 6.75) | 5.48 (2.35, 12.78) |  |
| **Years on ART** | 0.94 (0.88, 1.00) | 0.90 (0.84, 0.97) | F = 8.13, p < 0.01 |
| **Ever switched ART regimen** |  |  |  |
| Never switched | Ref | Ref | F = 10.50, p <0.01 |
| Ever switched | 1.96 (1.14, 3.37) | 2.75 (1.49, 5.10) |  |
| **Wald’s Test of adjusted odds ratio* | | | |
